# Peptidomimetics designed to bind to RAS effector domain are promising cancer therapeutic compounds

**DOI:** 10.1038/s41598-022-19703-6

**Published:** 2022-09-22

**Authors:** Chiara Pallara, Debora Cabot, Josep Rivas, Sonia Brun, Jesús Seco, Baraa Abuasaker, Teresa Tarragó, Montserrat Jaumot, Roger Prades, Neus Agell

**Affiliations:** 1 Iproteos S.L., Barcelona Science Park, Baldiri Reixac 10, 08028 Barcelona, Spain; 2 Department of Biomedical Sciences, Faculty of Medicine and Health Sciences, University of Barcelona, C/Casanova 143, 08036 Barcelona, Spain; 3 Institut d’Investigacions Biomèdiques August Pi i Sunyer (IDIBAPS), Barcelona, Spain

**Keywords:** Cancer, Cancer therapy, Targeted therapies

## Abstract

Oncogenic RAS proteins are important for driving tumour formation, and for maintenance of the transformed phenotype, and thus their relevance as a cancer therapeutic target is undeniable. We focused here on obtaining peptidomimetics, which have good pharmacological properties, to block Ras–effector interaction. Computational analysis was used to identify hot spots of RAS relevant for these interactions and to screen a library of peptidomimetics. Nine compounds were synthesized and assayed for their activity as RAS inhibitors in cultured cells. Most of them induced a reduction in ERK and AKT activation by EGF, a marker of RAS activity. The most potent inhibitor disrupted Raf and PI3K interaction with oncogenic KRAS, corroborating its mechanism of action as an inhibitor of protein–protein interactions, and thus validating our computational methodology. Most interestingly, improvement of one of the compounds allowed us to obtain a peptidomimetic that decreased the survival of pancreatic cancer cell lines harbouring oncogenic KRAS.

## Introduction

Ras genes were the first mutated genes identified in human cancers^1^ and the most recurrent (around 19% of tumours). Three genes named KRAS, HRAS and NRAS encode for the four RAS isoforms, KRAS being the most frequently mutated (75%) followed by NRAS (17%) and HRAS (7%). Pancreatic cancer has the highest incidence of RAS mutation of all cancers (88% of samples contain KRAS mutations)^2^.

RAS proteins participate in the regulation of cell proliferation, differentiation, survival and apoptosis^3^. They are small GTPases that cycle from the inactive (GDP-bound) to the active (GTP-bound) state, responding to extracellular signals due to their firm control by guanine nucleotide exchange factors -exchange factors (GEFs) and GTPase activating proteins (GAPs)^4–6^. Gly12, Gly13 and Gln61 are the most frequent oncogenic mutations in RAS, which retain the active state by inhibiting intrinsic GTPase activity or preventing the action of GAPs^7^. GTP-bound RAS interacts with different effector proteins, and therefore activates signal transduction pathways, with RAF/MEK/ERK and the phosphatidylinositol3-kinase (PI3K)/AKT pathways being the best known^8,9^.

All RAS isoforms have a highly conserved globular domain (residues 1–165), which contains: the catalytic lobe (with the guanosine nucleotide and effector binding sites, residues 1–86, almost 100% homology) and the allosteric lobe (residues 87–165); and the C-terminal domain, named the hypervariable region (HVR) (residues 166–188/9)^10^, which contains the membrane targeting signals and is not conserved among the different isoforms^11,12^.

Unfortunately, no efficient RAS therapy has been found to date. Targeting oncogenic RAS has been challenging due to the difficulty in designing drugs to recover the GTPase activity and the lack of classical “druggable pockets”^10,13,14^. In fact, the only drugs specifically targeting oncogenic RAS that have reached the clinic are those directed against the mutant KRASG12C, which is mainly found in lung cancer^15–17^. Thus, different strategies have been developed to try to inhibit RAS mutants indirectly, which can be summarized as follows: a) focused on inhibiting RAS localization and spatial assembling to the plasma membrane; b) by inhibiting downstream effectors of RAS that are more easily targeted by drugs: c) by blocking synthetic lethal oncogenic RAS interactors; and d) by interfering with the RAS-dependent metabolism of cancer cells (reviewed in^10,13,14,18^).

The unclear results obtained using these experimental approaches has led the scientific community to refocus on the search for direct RAS inhibitors^19^. With this objective, two strategies are currently being followed: the first involves searching for molecules that block the interaction with non-effector RAS proteins that bind to the HVR and/or the allosteric lobe and which modulate RAS activity; and the second is looking for drugs that inhibit the binding of RAS with its effectors^20,21^. In this context, Rigosertib, a compound that binds to the Ras-binding domain of multiple RAS effectors, has proven to inhibit tumour growth in KRAS-mutant colon and pancreatic cancer mouse models^22^. Unfortunately, a randomized phase 2/3 trial found no benefit from the addition of rigosertib to gemcitabine in patients with previously untreated metastatic pancreatic cancer^23^.

In this manuscript, we focus on the second approach to generate a compound for RAS therapy. In fact, the modulation of protein–protein interactions (PPIs) has gained much attention in the scientific community in recent years due to the large number of PPIs involved in the cellular machinery^24^. However, the modulation of PPIs is highly challenging because of the nature of these interactions, which usually take place in flat and large patches of the protein surface with no clefts where a small molecule can properly interact and accommodate to hamper a PPI^25^. An alternative to the use of small molecules for the modulation of PPIs is the use of biologics, such as antibodies. These, which are considered large structures rather than small molecules, have the capacity to exquisitely recognise and interact with large protein surfaces. However, these large entities do not have the capacity to cross biological barriers, which limits their therapeutic use, given that many PPIs take place inside the cell. In this scenario, the use of peptides has emerged as a promising tool to modulate the biological activity of PPIs^26^. Peptides are found in the middle of the chemical space between traditional small molecules and antibodies, being able to recognize and bind, in a specific manner, to protein patches and to cross biological barriers when properly engineered, *e.g.* introducing non-natural amino acids, cyclization or having unusual peptide bonds^27^. Peptides with these modifications are usually known as peptidomimetics.

We used a computational approach to design a peptidomimetic able to disrupt the interactions of RAS proteins with their effectors. The workflow was as follows: (1) the identification of RAS pharmacophore sites, (2) virtual screening of a library of 80,000 tri- and tetra-peptidomimetics against the identified hot spots of the protein, (3) in silico prediction of the cell membrane permeability of the identified hits, and (4) synthesis and in vitro evaluation of the most promising candidates. This approach allowed us to obtain, in a quick and cost-effective manner, one compound (named here P1.3) that has the capacity to efficiently inhibit the interaction of RAS with its effectors in cells in vitro and inhibits survival of pancreatic tumour cells expressing oncogenic KRAS.

## Results

### Structural characterization of RAS–effector complexes

**Figure 1:**
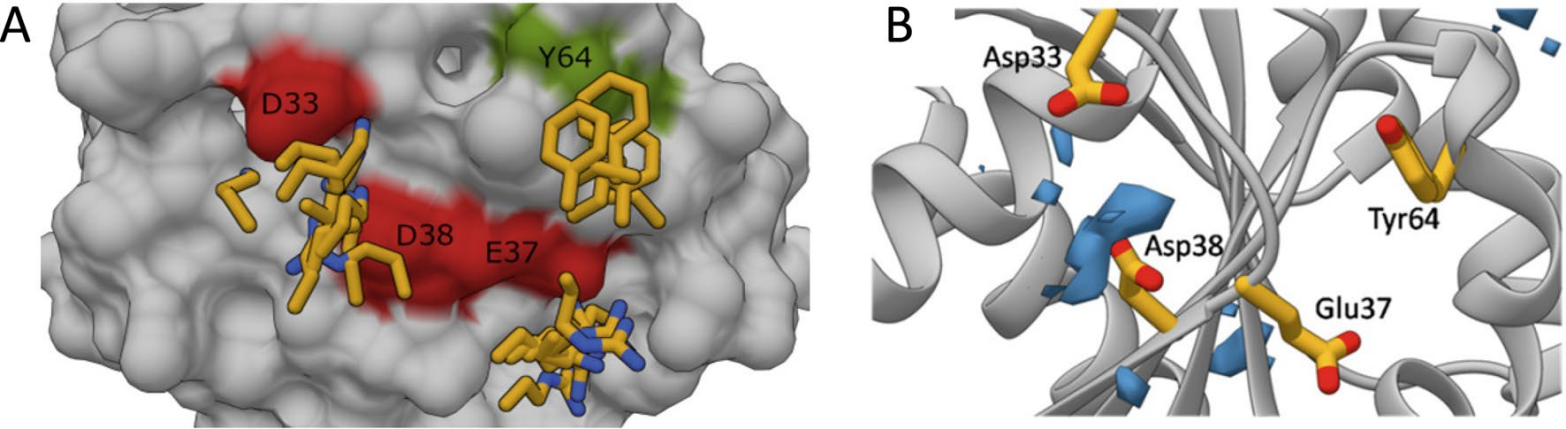
Peptidomimetic design. (**A**) Superimposition of eight structures of RAS GTPase in complex with effector proteins, including CRAF (PDB code 3KUD), BYR2 (PDB code 1K8R), ARAF (PDB code 2MSE), RALGDS (PDB code 1LFD), PI3K (PDB code 1HE8), PLCε (PDB code 2C5L), RASSF (PDB code 3DDC), and GRAB14 (PDB code 4K81). Highly conserved residues among RAS effector proteins are underlined with yellow bars. RAS protein surface is coloured in grey, residues involved in intermolecular contacts with highly conserved residues among effector proteins are underlined in red (Asp33, Glu37, Asp38) and green (Tyr64). (**B**) Substrate-binding site mapping on effector binding surface of RAS protein. The positively charged high propensity region between Asp33 and Asp38 is shown as a blue surface. RAS residues involved in intermolecular contact with highly conserved effector protein residues are underlined with yellow bars (Asp33, Glu37, Asp38 and Tyr64). Images were drawn using the docking program indicated in “Methods”.

The identification of RAS pharmacophore sites was performed by the structural comparison of the RAS-GTPase in complex with several effector proteins. To date ten structures of RAS protein in complex with its effectors have been determined (Table S1). In order to map the atomic interactions responsible for RAS–effector binding, we extracted information about the interacting interface from all these complex structures and combined this with their sequence alignments. Interestingly and as previously shown by Nakhaeizadeh and colleagues, although RAS effectors show low overall sequence similarity, their mode of interaction appears to be well conserved, as can be seen after the superposition of the complex structures on the RAS structure^28^. But even more interestingly, we did identify some key residues on the RAS protein surface, namely Asp33, Glu37, Asp38, and Tyr64, involved in intermolecular contacts with specific residues that are highly conserved among virtually all the effector proteins (Fig. 1A, Table 1).

**Table 1:** Intramolecular contacts conserved among RAS-effectors complexes.

RAS	RAS-effectors							
	PI3K	BYR2	RALGDS	PLCε	ARAF	RASSF	CRAF	GRAB14
	(1HE8)	(1K8R)	(1LFD)	(2C5L)	(2MSE)	(3DDC)	(3KUD, 4G0N, 4G3X)	(4K81)
Glu37	Lys223	Arg74	Arg20	Asn2140	Arg819	Lys236	Arg59	Lys111
Asp38	–	Lys101	–	Lys2171	Lys836	Lys283	Lys85	Arg120
Asp33	Lys251	Lys101	Lys52	Lys2171	–	Lys283	Lys85	Lys140
Tyr64	Phe221	Ile72	Ile18	Phe2138	–	Phe234	–	Val109

### Analysis of the substrate-binding site based on mixed-solvent molecular dynamics (MixMD) simulation

A molecular dynamics simulation of RAS protein crystal structure in mixed solvent was performed in order to identify predominant protein surface regions responsible for the interaction with potential ligand compounds on the effectors’ binding interface. During the MixMD simulation, a mixed solvent box composed by benzene, propane, ethanol, propionic acid (negatively charged) and ethylamine (positively charged) organic probes properly combined with TIP3P water was used. A region with high propensity for positive charges was found in a relatively exposed cavity surrounded by the negatively charged side chains of Asp33_RAS_ and Asp38_RAS_ residues (Fig. 1B). In that regard, during calculations, ethylamine molecules spend significantly more time around these two residues than in other solvent exposed surface regions. Interestingly, this corresponds to the area where the positively charged residues that are highly conserved among the effector protein sequence (*i.e.*, Lys251_PI3K_, Lys 101_BYR2_, Lys52_RALGDS_, Lys2171_PLCε_, Lys283_RASSF_, Lys85_CRAF_) were found to cluster. In contrast, no hydrophobic or positively charged contours (benzene, propane) were detected around Tyr64_RAS_ or Glu37_RAS_.

Based on the results obtained from both the analysis of RAS protein crystal structures and MD simulation, the Asp33_RAS_ and Asp38_RAS_ residues were selected as the substrate binding site since they were identified by both analyses. These data were subsequently used in virtual screening for putative RAS inhibitors, both to adapt the composition of the peptidomimetic library used as ligands (such as in the hit identification step*)* as well as to set the position and the size of the docking box (in both the hit identification and hit optimization step). Thus, as described in the “Methods”, a peptidomimetic library including only positively monocharged compounds was selected and the docking box was fixed around the Asp33 and Asp38 RAS protein residues.

### Virtual screening of RAS inhibitors

Virtual screening of chemical databases through molecular docking protocols can help find novel potential leads suitable for further development^29,30^. In this work, two molecular docking experiments were carried out using the SMINA docking program to firstly identify and then optimize novel inhibitors for RAS protein.

**Figure 2:**
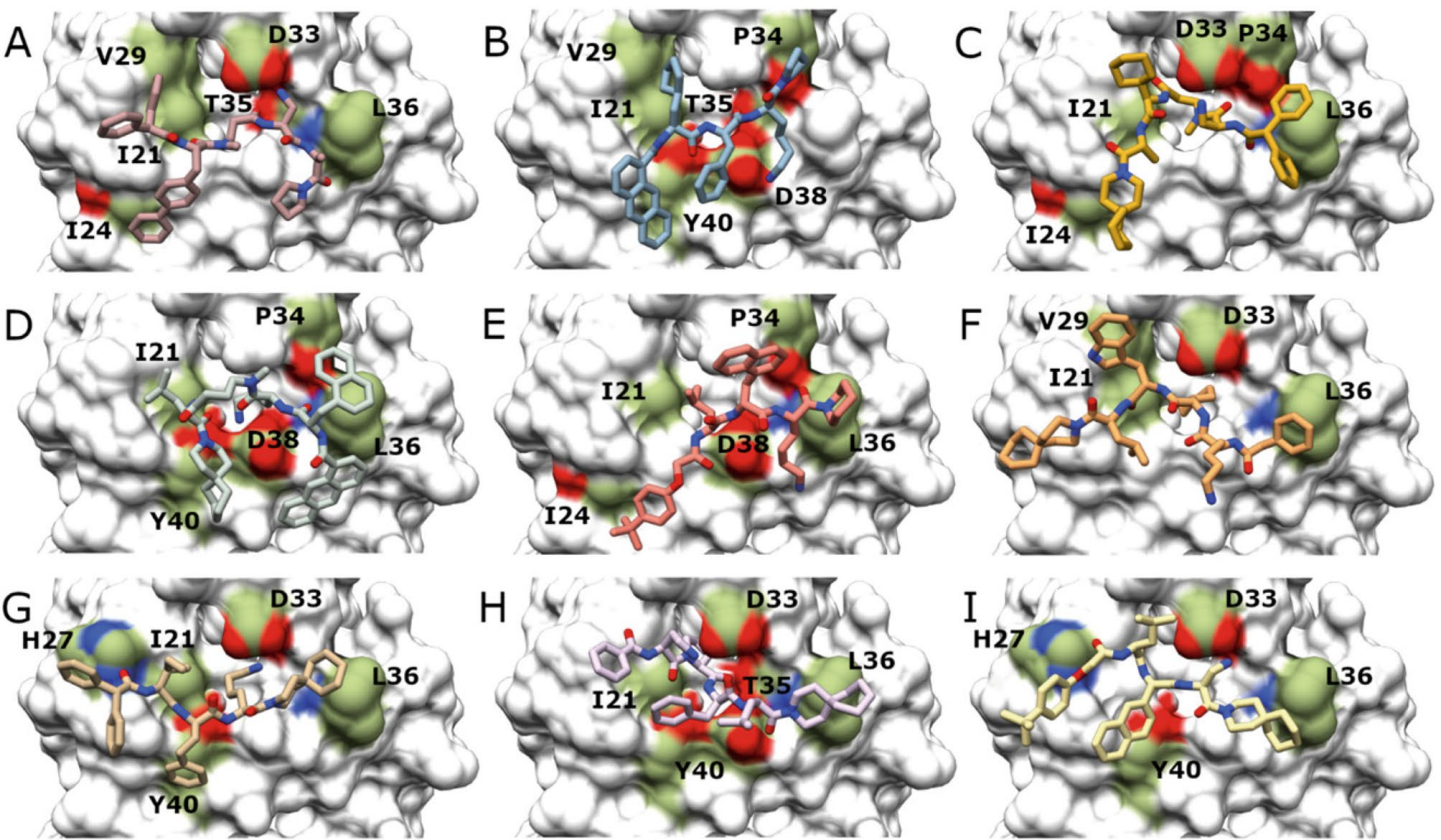
Peptidomimetic characteristics. In silico predicted binding modes of (**A**) P1, (**B**) P2, (**C**) P3, (**D**) P4, (**E**) P5, (**F**) P6, (**G**) P7, (**H**) P8 and (**I**) P9. Contact point residues on the RAS surface (PDB code 5P21) are shown in colour on the RAS protein surface: aliphatic areas are shown in green, areas with amino groups in blue and areas containing carboxylic moieties in red. RAS residues involved in contact with the compounds are named using the one letter amino acid code; the residue number is shown next to the letter. Images were drawn using the docking program indicated in “Methods”.

A library of 80,000 tri- and tetra-peptidomimetics, containing at least one positively charged and both natural and non-natural amino acids in their structure was generated in silico*.* All of the first peptidomimetic dataset was docked into the RAS effectors binding region around the substrate binding site previously identified; these were subsequently ranked according to the corresponding docking energy and finally filtered on the basis of their geometric and energetic properties (as described in the “Methods”). After filtering, a total of nine compounds were selected based on their favourable binding stability to RAS protein and cell membrane permeability as well as their optimal overall binding mode (Table 2). As shown in Fig. 2, the nine hit compounds share similar binding modes.

**Table 2:** First set of inhibitors, docking scores and PASA.

Compound	Formula	Structure	Docking score (Kcal/mol)	PASA (Å^2^)
P1	C_43_H_51_N_6_O_5_		− 10.2	128
P2	C_42_H_52_N_5_O_4_		− 9.7	93
P3	C_42_H_61_N_6_O_5_		− 9.6	105
P4	C_54_H_69_N_6_O_5_		− 9.1	123
P5	C_42_H_60_N_5_O_5_		− 8.9	111
P6	C_49_H_72_N_7_O_5_		− 8.4	116
P7	C_46_H_64_N_5_O_4_		− 8.4	86
P8	C_41_H_59_N_6_O_5_		− 8.3	133
P9	C_44_H_62_N_5_O_5_		− 8.0	104

### Effect of peptidomimetics on Ras signalling

**Figure 3:**
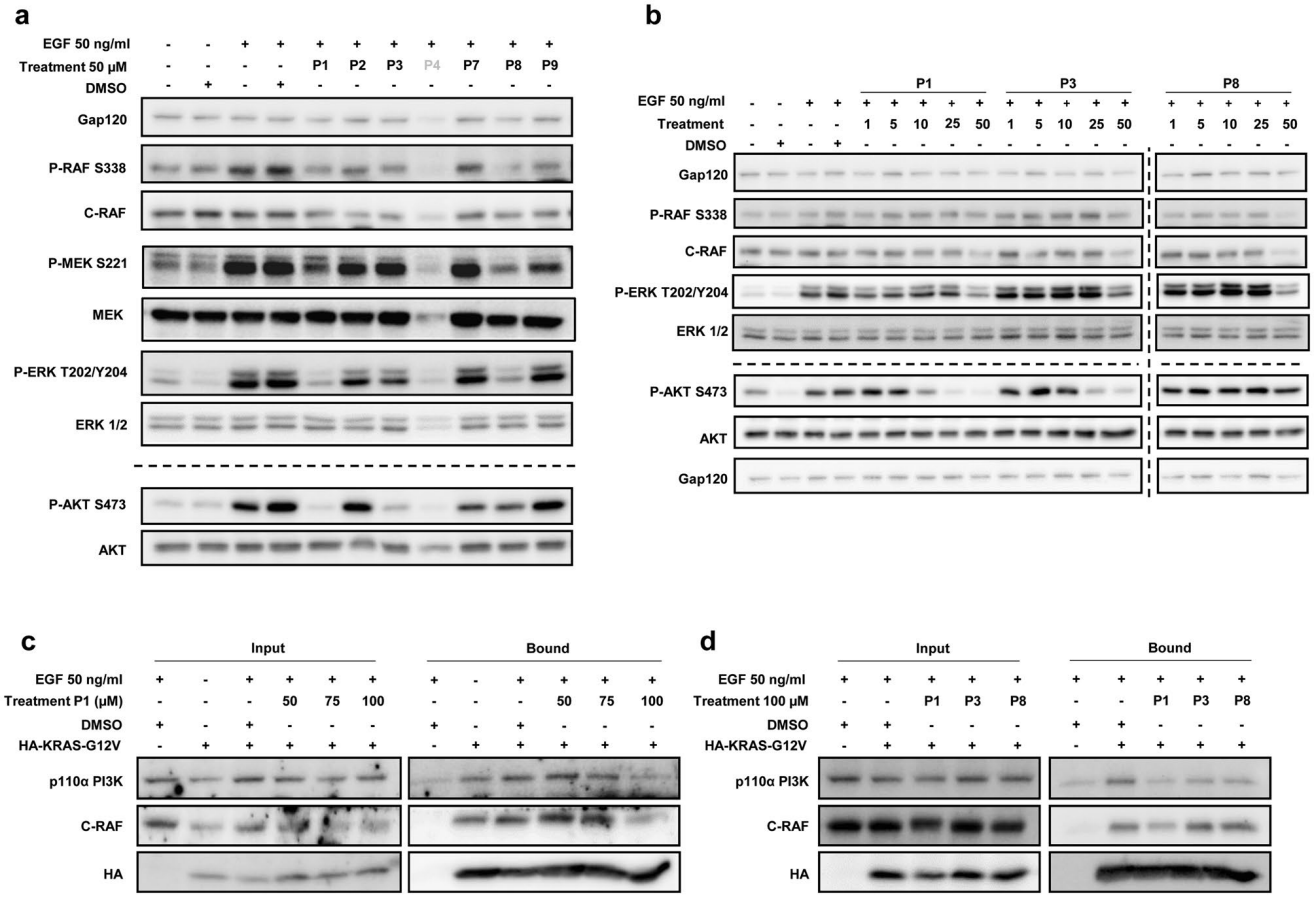
P1 diminishes endogenous downstream RAS signalling and disrupts KRAS interaction with the effectors c-RAF and PI3K. (**a**) hTERT-RPE starved cells were incubated with 50 µM of the indicated peptidomimetics for 2 h and the levels of activation of the RAF/MEK/ERK and PI3K/AKT pathways after EGF stimulation for 10 min were studied by Western blot. Specific antibodies against the active phosphorylated and total proteins were used. GAP120 was utilized as a loading control. (**b**) The same analyses were performed as in (**a**), but incubating the cells with the indicated peptidomimetics in a range from 1, 5, 10, 25 and 50 µM. (**c**) Co-immunoprecipitation of HA-KRASG12V with C-RAF or with PI3K was analysed in starved HeLa cells expressing HA-KRASG12V after being incubated with P1 (in a range from 50, 75 and 100 µM) for 2 h, and EGF-stimulated for 10 min. IP was performed with anti-HA antibodies and Western blot of the bound and input fractions with anti-p110αPI3K and anti-C-RAF. (**d**) The same analyses as in (**c**) were done, but incubating the cells with 100 µM of the indicated peptidomimetics. All experiments were repeated at least three times.

In order to investigate putative biological effects of the nine peptidomimetics designed to bind to the RAS effector domain, we first analysed the ability of these compounds to prevent RAS downstream signalling by EGF activation. P5 and P6 were discarded because of their very low solubility. hTERT-RPE serum-starved cells were initially treated with the peptidomimetics (50 µM) for 2 h before being stimulated with EGF for 10 min. After that, the levels of activation of the RAF and PI3K pathways were assessed by Western blot with the corresponding antibodies against the phosphorylated active forms of c-RAF, MEK, ERK and AKT. Data show that P1 and P8 were able to attenuate the activity of RAF, MEK and ERK more efficiently than P3, whereas the effect of P2, P7 and P9 was milder. Regarding the PI3K pathway P1 and P3 induced a higher reduction of AKT activation (S473-P) than P7 and P8, while P2 and P9 had no significant effect. Circumstantially, we could not obtain any quantifiable amount of protein in lysates from cells treated with P4 probably due to high cell death (Fig. 3a). These results encouraged us to compare the inhibitory capacity of P1, P3 and P8 at lower concentrations. The best candidate for inhibiting PI3K downstream signalling was P1 since a reduction in the levels of AKT phosphorylation (S473 and Thr308) was detected even at 10 µM. However, all three peptidomimetics had to be at the highest concentration assayed to decrease ERK activation (Fig. 3b). In conclusion, P1 was found to be the best peptidomimetic to diminish RAS signalling through the PI3K/AKT and c-RAF1/MEK/ERK pathways in non-transformed cells.

### Oncogenic KRAS–effector protein–protein interactions in vivo are impaired by treatment with the peptides

Since the peptides were designed in silico to interact with the RAS effector domain and the treatment of cells with these compounds (specifically P1, P3 and P8) decreased RAS downstream signalling via RAF and PI3K, we evaluated the possibility that the compounds were blocking RAS–effector binding and thus inhibiting activation of the pathways. An immunoprecipitation assay was devised by transfecting HeLa cells with the pEF-HA-KRASG12V plasmid before treatment with the peptides and EGF activation. First, we evaluated the effect of P1 on the interaction of HA-KRASG12V with c-RAF and PI3K by subjecting the cells to increasing concentrations of P1 (from 50 to 100 µM). Interestingly, the data showed that P1 treatment reduced KRAS binding to both effectors at 100 µM compared with the control (DMSO) (Fig. 3c, and S1). We thus proceeded to test the effect of P3 and P8 under the same conditions. The experiment revealed that while P1 treatment reduced KRAS-G12V (always GTP-loaded) binding to both effectors, P3 and P8 disrupted only the interaction with PI3K (Fig. 3d).

Biological assays conducted (Fig. 3) have demonstrated that the treatment of cells with the peptidomimetic P1 reduced oncogenic KRAS interaction with its effectors PI3K and c-RAF, which negatively affected downstream RAS signalling. This led us to select P1 as the peptidomimetic hit for further in silico optimization to improve its inhibitory potency.

### Hit optimization

As in the hit identification step, all the P1-derived compounds were docked into the RAS–effectors binding region using the same protocol as described above. Finally, a total of four compounds were selected bearing favourable binding stability to the RAS protein and cell membrane permeability as well as an overall similarity in binding mode to P1 (Table 3).

**Table 3:** Second set of inhibitors, docking scores and PASA.

Compound	Formula	Structure	Docking score (Kcal/mol)	PASA (Å^2^)
P1.1	C_50_H_66_N_6_O_6_		− 10.5	114
P1.2	C_51_H_58_N_6_O_5_		− 10.2	75
P1.3	C_47_H_58_N_6_O_5_		− 9.6	79
P1.4	C_49_H_59_N_5_O_6_		− 9.6	91

**Figure 4:**
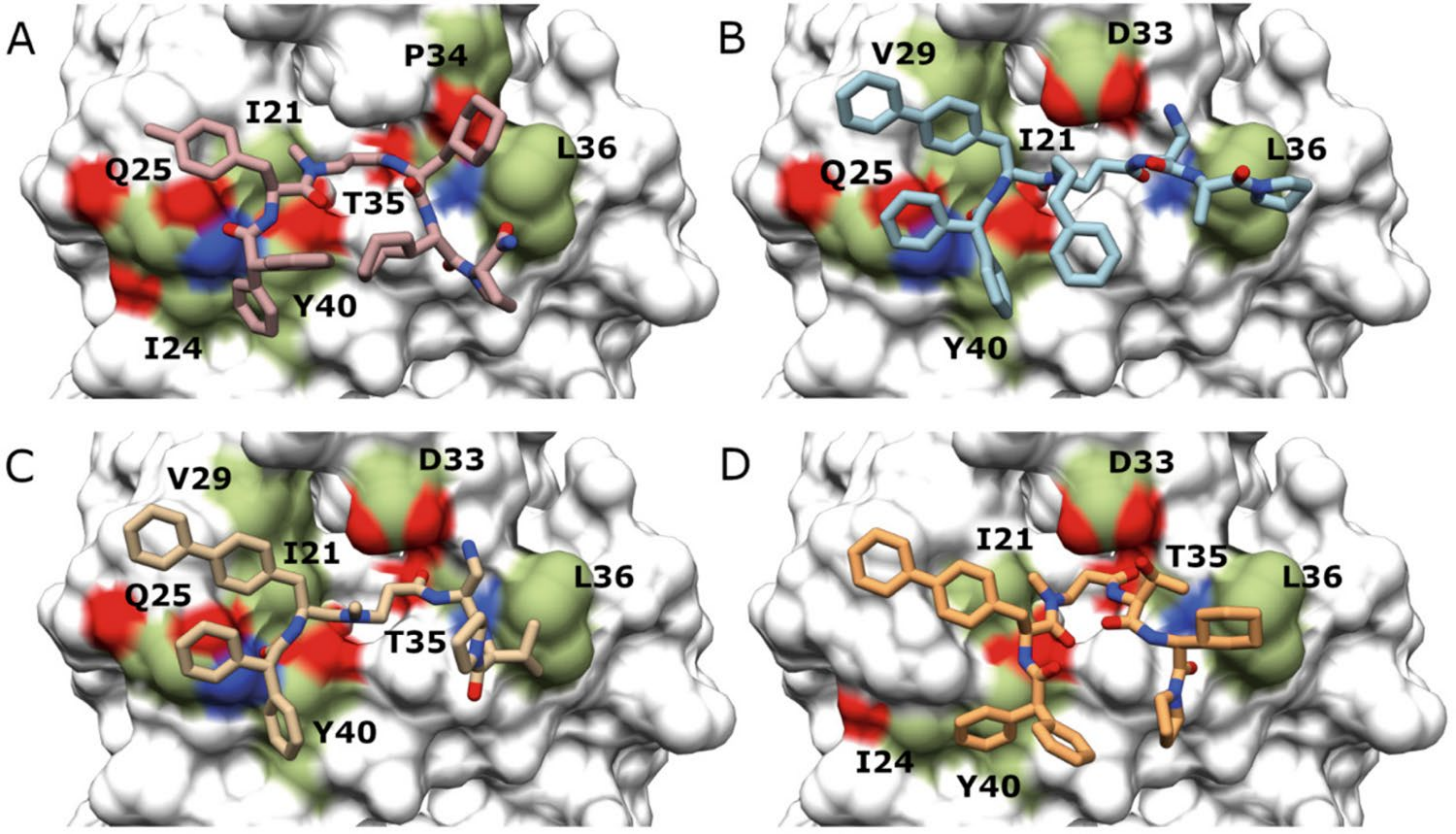
Peptidomimetic in silico improvement. In silico predicted binding modes of (**A**) P1.1, (**B**) P1.2, (**C**) P1.3 and (**D**) P1.4. Contact point residues on the RAS surface (PDB code 5P21) are shown in colour on the RAS protein surface: aliphatic areas are shown in green, areas with amino groups in blue and areas containing carboxylic moieties in red. RAS residues involved in contact with the compounds are named using the one letter amino acid code; the residue number is shown next to the letter. Images were drawn using the docking program indicated in “Methods”.

As shown in Fig. 4, the four P1-derived compounds (P1.1, P1.2, P1.3 and P1.4) share similar binding modes.

### Peptidomimetic P1.3 negatively affects downstream RAS signalling, effector binding capacity and cancer cell viability

**Figure 5:**
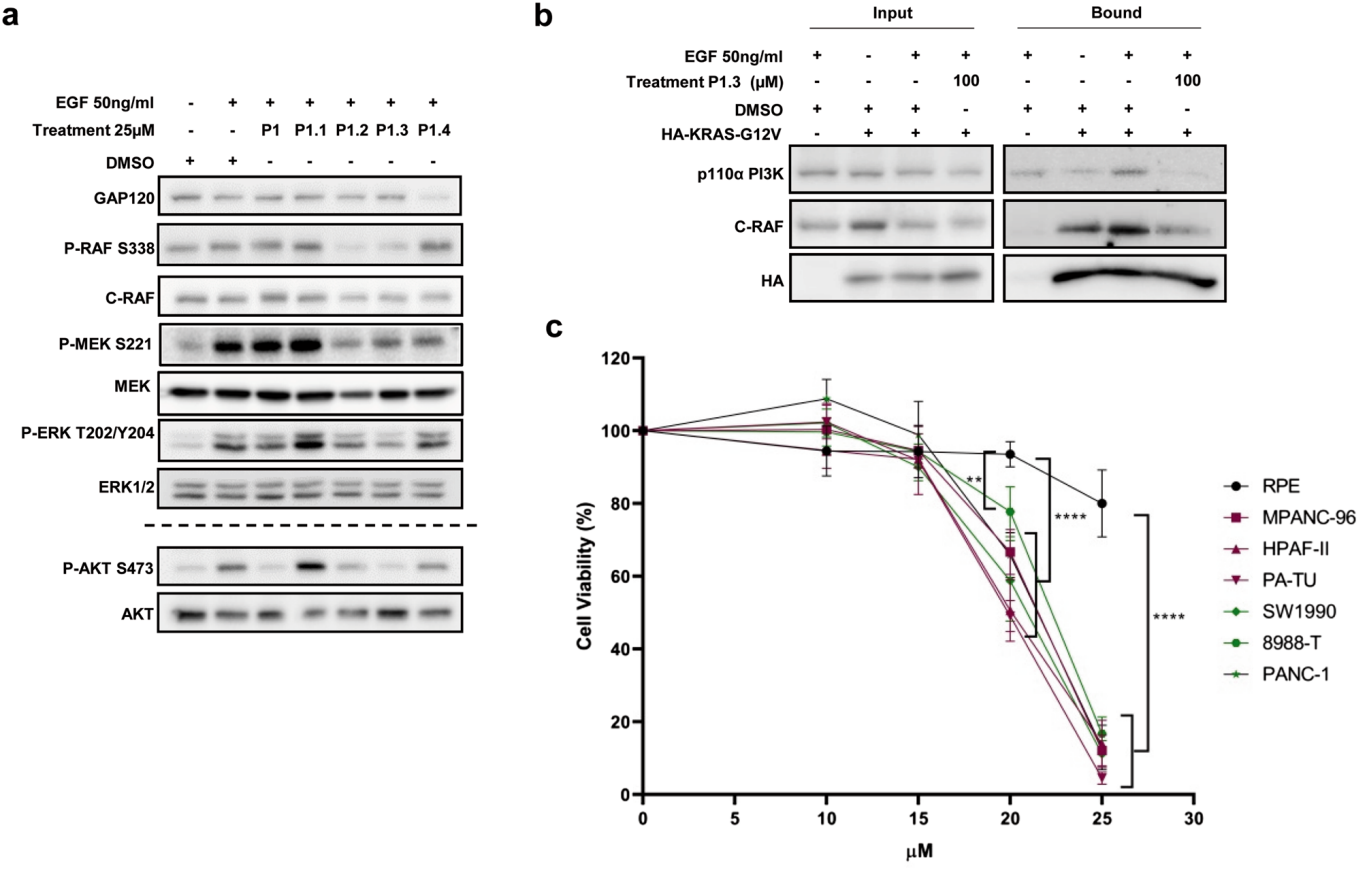
P1.3 reduces PI3K and c-RAF1 signalling and interaction with KRAS; and decreases pancreatic tumour cells but not normal cells viability. (**a**) hTERT-RPE starved cells were incubated with 25 µM of the indicated peptidomimetics for 2 h and the levels of activation of the RAF/MEK/ERK and PI3K/AKT pathways after EGF stimulation for 10 min were studied by Western blot. Specific antibodies against the active phosphorylated and total proteins were used. GAP120 was utilized as a loading control. (**b**) Co-immunoprecipitation of HA-KRASG12V with C-RAF or with PI3K was analysed in starved HeLa cells expressing HA-KRASG12V after being incubated with 100 µM of P1.3 for 2 h, and EGF-stimulated for 10 min. IP was performed with anti-HA antibodies and Western blot of the bound and input fractions with anti-p110αPI3K and anti-C-RAF. (**c**) Effect of P1.3 on the cell viability of six pancreatic adenocarcinoma human cell lines (all harbouring oncogenic KRAS mutations) and of a non-transformed cell line (hTERT-RPE). Cells were treated with P1.3 in a dose range from 0 to 25 µM and incubated for 24 h, when cell viability was determined by MTS assay. Experiments (**a**) and (**c**) were repeated at least three times, and (**b**) once. Differences were assessed using one-way ANOVA and Tukey Multiple Comparisons Test and considered significant when *p* ≤ 0.05.

The ability of the new peptidomimetics P1.1, P1.2, P1.3 and P1.4 (derived from P1) to inhibit RAS signalling was analysed as in Fig. 3. At 25 µM, both P1.2 and P1.3 were able to reduce the levels of P-RAF, P-MEK, P-ERK and P-AKT, but P1.3 turned out to be the most efficient. On the contrary, P1.1 and P1.4 had no expected effect (Fig. 5a). RBD-pull down analysis confirmed that the levels of RAS-GTP were not modified by P1.3 treatment (Fig. S2), in agreement with the fact that the action of the peptidomimetic was to inhibit the interaction of RAS with the effectors. Concomitantly, P1.3 disrupted the interaction between KRAS-G12V (always GTP-loaded) and c-RAF and PI3K (Fig. 5b) in the same conditions as in Fig. 3. Finally, surface plasmon resonance analysis was performed to confirm direct interaction between KRAS and P1.3. The affinity constant (KD) of P1.3 for GTP-loaded KRAS was 15.7 µM (Fig. S3), while no stable sensorgrames could be obtained when analysing interaction between GDP-loaded KRAS and P1.3. These data further confirmed that, according with the initial design, P1.3 was directly interacting with the effector domain of Ras in its GTP-loaded conformation.

Based on these results, we performed viability assays with six pancreatic adenocarcinoma human cell lines (PDAC) harbouring different oncogenic KRAS mutations and with a non-transformed cell line, all of them epithelial. Exciting results from these dose–response experiments (from 0 to 25 µM) at 24 h (Fig. 5c) showed that P1.3 was able to reduce cell viability with an IC_50_ of approximately 20 μM in all tumour cell lines, but at these concentrations only affected less than 5% of normal cells.

Although the specific mechanism of cell death induction of pancreatic cells remains to be analysed, from these biological studies, we can conclude that treatment with the peptidomimetic P1.3 kills pancreatic tumour cells expressing oncogenic KRAS, with a negligible effect in normal cells.

## Discussion

Since oncogenic RAS proteins are not only important for driving tumour formation but also for maintenance of the transformed phenotype, both in tumour cell models in vitro^31,32^ and in mouse models in vivo^33–38^, their relevance as cancer therapeutic targets is undeniable^39,40^. Additionally, the effectiveness of inhibiting RAS signalling by blocking RAS–effector PPIs has been proven using intracellular antibodies^41^, and by the development of multivalent small-molecule Pan-RAS inhibitors^42^.

Here, with the aim of obtaining new compounds with the capacity to block the interaction of RAS with their effector proteins, and with potential pharmacological properties, we used engineered peptides, namely peptidomimetics, to modulate these PPIs. To achieve this objective, we used a combination of computational tools to accelerate the drug discovery process. In this computational approach, we considered the intrinsic flexibility of peptides using the Ramachandran plot^43^ to exclude compounds with unfeasible conformations from exhaustive calculations in order to reduce computing time. Commercial docking software is intended mainly for small molecule (rigid conformation) calculations, and when used for flexible and large molecules the accuracy of the calculations dramatically decreases^44^. By adapting the existing docking software SMINA to Ramachandran dihedral angle requirements we were able to exclude potential false positive candidates from the screening process, thus optimizing the use of the available computational resources. In addition to this, we also applied an in silico permeability filter to those candidates with better docking scores. This filter is based on the calculation of the topological polar surface area (PASA) of the molecules. PASA can be used as an indicative parameter of the capacity of the molecule under study to have a “chameleonic” behaviour^45,46^ in hydrophobic environments, such as the cell membrane. Using this parameter, we can estimate if a molecule could be soluble or partially soluble in aqueous media but also be hydrophobic enough to be able to cross the cell membrane by passive diffusion. Only compounds with a PASA value below 130 Å_2_ were selected for synthesis. This threshold was calculated from *in house* experiments (data not published) in which we found that peptidomimetics having this PASA value or lower have good permeability by passive diffusion.

After this computational process, nine of the 80,000 compounds contained in the virtual library were selected for synthesis and in vitro evaluation. Since the binding site of the peptidomimetics was not dependent on oncogenic RAS mutations, human non-transformed epithelial cells treated with EGF were used for the initial evaluation of the impact of the compounds on RAS signalling. The analysis using tumour cells would have been more puzzling due to the presence of strong negative feedback loops and to the high context dependency on this signal transduction pathway in tumour cells^47,48^. Two out of the nine candidates (P5 and P6) were not evaluated because of their lack of solubility in the cellular media used for the in vitro efficacy study. For the remaining candidates, compounds P1, P3 and P8 were able to decrease RAS downstream signalling, compound P1 being the most effective. This specific compound had the best docking score in the in silico calculations (− 10.2 kcal/mol, Table 1). Following compound synthesis and evaluation, it is worth highlighting that a high percentage of the tested compounds showed in vitro efficacy, which indicates the high accuracy and predictability of the in silico process used in this study.

In a second step of the drug discovery process, compound P1 was used as a scaffold for further compound refinement. Here, a rational design was used to propose new analogues for P1 that were afterwards evaluated in silico, as for the preceding candidates (P1–9), to select the best candidates for synthesis. After this process, four candidates were selected for synthesis and in vitro evaluation. Compound P1.1 was designed to increase the surface of the compound in order to increase the number of contact points between the molecule and the target protein. In this regard, the alanine moiety was replaced by cylohexyl glycine and the amino propinoic acid moiety by cyclohexyl alanine. To balance the increase in the hydrophobicity of this analogue, a proline carboxylamide was placed on the *C*-terminal of the molecule and the *p*-phenyl-pehnylalanine moiety was replaced by *p*-methyl-phenylalanine. In compound P1.2, the *N*-methyl group on the side chain of the β-alanine moiety was replaced by a longer carbon chain attached to an aromatic group, propylbenzene. The idea behind this modification was to increase the overall hydrophobicity of the compound (to increase permeability) and increase the n-alkyl shielding capacity. A 4-carbon chain, instead of a methyl, would give a certain degree of flexibility, allowing the 6-carbon aromatic ring to wrap the molecule, thus facilitating the chameleonic behaviour of this candidate. Moreover, an increase in the number of contacts with the target protein surface was expected. For compound P1.3, an alanine was substituted by isoleucine and *N*-methyl groups were selectively added to the backbone of the compound. These modifications were intended to slightly increase the number of contact points with the protein while preserving the binding mode of P1. Finally, in compound P1.4, alanine was substituted by cyclohexyl glycine to increase the number of contacts with the target protein. The amino propionic acid moiety was replaced by threonine to preserve the hydrogen bond donor on the molecule but also slightly increase the number of contact points with the molecule. The compounds P1.2 and P1.3, the most conservative analogues of P1, were effective at inhibiting the RAF/MEK/ERK and PI3K/AKT signalling pathways. Lack of positively charged groups in P1.1 and P1.4 could give a lower interaction with the negative residues of the RAS interaction area (Asp33 and Asp38) and this could explain its lower effect on RAS signalling. In fact, these two peptidomimetic could be considered as negative controls.

According to the intended function of the peptidomimetics and in correlation with the detected inhibition of the signal transduction pathways, disruption of the interaction of KRAS with two of its effectors, c-RAF and p110-PI3K, was observed with the compounds P1 and P1.3.

The higher effect of P1.3 on cell viability in PDAC cells compared with non-transformed epithelial cells is of major relevance for cancer therapy. Ablation of HRAS, NRAS and KRAS in fibroblasts in culture causes growth arrest^49^, and thus the low toxicity in non-transformed cell lines is surprising. Therefore, it is possible that the peptidomimetic might not completely inhibit the interaction of RAS with its effectors. In this study we used c-RAF and PI3K as markers of the activity of the peptidomimetics, but the peptidomimetics could impair the interaction of RAS with any other of the already described effectors^1^ or with still unknown effectors. Consequently, the major effect of P1.3 on the viability of PDAC cells compared to non-transformed cells may be explained either by a higher dependency of PDAC cells on a decrease in ERK or AKT activity or by the specific dependency of these cells on another RAS effector. Surprisingly, our preliminary studies also show that A375 cells (with oncogenic BRAFV600E) are also sensitive to P1.3. This may seem surprising, since these cells have been used as representative cells refractory to RAS inhibition, but the inhibitors used in these studies, in contrast to ours, are specific for an oncogenic RAS mutation or RAS isoforms^50,51^. The dependency on the wild type RAS isoforms to support PI3K/AKT is a common event in tumour cells harbouring oncogenic mutations in RAS/RAF/ERK pathway^52^. In fact, A375 cells are highly sensitive to PI3K/mTOR inhibitors^53^. Thus, our compound, by inhibiting all isoforms could be reducing also survival of cells harbouring BRAFV600E mutations.

In this study, we demonstrated that the computational design of peptidomimetics is a good strategy to obtain RAS inhibitors with a potential therapeutic role. To date, several cycle and bicycle peptides have been developed to occupy the effector-binding region of RAS^54^, thereby inhibiting Ras signalling. But the low molecular weight and the simple structure of the peptidomimetics that we have described give them some clear advantages as potential drugs in comparison with these cycle and bicycle peptides: they are easy to synthesize, and thus more economic to produce; they probably have the best ADME properties; and, finally, they are probably less immunogenic.

Interestingly, we obtained a peptidomimetic that inhibits the survival of PDAC cells while not being toxic for non-transformed cell lines. Therefore, it could be a lead compound in the search for drugs to treat metastatic PDAC, currently an incurable disease.

## Methods

### Synthesis of peptidomimetics

All compounds were synthesized by means of solid-phase peptide synthesis (SPPS) following an Fmoc/*t*Bu strategy. Syntheses were performed on a 100 µmol-scale/each using the 2-chlorotrytil resin (except for P1.1, for which the H-Rink amide chemmatrix was used). Syntheses were carried out manually in polypropylene syringes fitted with a porous disk at the bottom. While growing the peptide chain intermittent manual stirring was carried out to ensure the proper mixing of the reagents. Solvents and soluble reagents were removed by suction. Amino acid couplings (either L-, D- or non-natural) were performed using 4 equivalents of the Fmoc-protected amino acids, 4 equivalents of 2-(1H-Benzotriazole-1-yl)-1,1,3,3-tetramethylaminium tetrafluoroborate (TBTU) and 8 equivalents of *N,N*-diisopropylethylamine (DIEA) in dimethylformamide (DMF) (1 × 75 min). The extent of the reaction was monitored using either the Kaiser test (primary amines)^55^ or the Chloranil test (secondary amines)^56^. In those cases in which the coupling was not fully accomplished, a recoupling step was performed using the standard coupling conditions. The Fmoc group was removed from the amino acids (once the coupling reaction was successfully completed) using a mixture of 20% of piperidine in DMF (2 × 1 min and 1 × 10 min).

Selective *N*-alkylation of the compound backbone was performed using the method described by Miller et al.^57^, which is divided into the following three steps (these steps are performed after Fmoc removal of the amino acid anchored onto the resin that is going to be *N*-alkylated): (1) protection and activation of the amino group with *otro*-nitrobenzensulfonate (*o*-NBS): 4 equivalents of o-NBS, 3 equivalents of 2,3,5-Collidine in DMF (1 × 30 min and 2 × 20 min), (2) deprotonation and *N*-alkylation: 3 equivalents of 1,8-Diazabicyclo[5.4.0]undec-7-ec in DMF are added to the resin (5 min), after that 10 equivalents of desired alkylsulfate are added to the resin (10 min). This treatment is repeated twice. (3) *o*-NBS removal: two treatments with a mixture of 10 equivalents of β-mercaptoethanol and 5 equivalents of 1,8-Diazabicyclo[5.4.0]undec-7-ec in DMF are performed (1 × 10 min and 1 × 40 min).

### Molecular modeling

#### Structural analysis of RAS–effector complexes

To date, 10 Ras–effector complex structures have been reported. They were downloaded from the RCSB PDB database (http://www.rcsb.org), were aligned on the RAS structure using Chimera software^58^ and all the crystallographic waters were removed. Finally, the key interacting residues were defined as those involved in intermolecular contacts (defined as pairs of residues with a distance of 4.0 Å between the effector and RAS proteins) and conserved in virtually all of the complexes.

#### Mixed-solvent molecular dynamics (MixMD) simulation on RAS GTPase protein

The crystal structure of RAS GTPase protein (PDB code 5P21^59^) was downloaded from the RCSB PDB database (https://www.rcsb.org/) and used as the input structure to perform a 50-ns long explicit mixed-solvent molecular dynamics (MixMD) simulation.

In the first preparatory step, the co-crystallized GppNHp molecule was replaced by the GTP original cofactor, whose parameter files were downloaded from the AMBER parameters database (http://research.bmh.manchester.ac.uk/bryce/amber/). Thus, the overall system was properly protonated and placed in a periodic cubic mixed solvent box composed of benzene, propane, ethanol, propionic acid and ethylamine organic probes properly combined with TIP3P water molecules (the minimum distance between protein and edges of the box was set at 10 Å). The overall protonation, solvation, and parameterization of the system was performed using the *Leap* module of AmberTool16^60^ and using the ff12 AMBER force field.

As detailed below, a two-step conjugate gradient-based minimization was run followed by a four-step equilibration and a 50-ns long MD simulation using the NAMD simulation package^61^. The SHAKE algorithm^62^ and Particle Mesh Ewald (PMD) method^63^ were applied (to restrain all bonds to hydrogen atoms and compute long-range Coulomb interactions, respectively) in all simulations. A time step of 2 fs and a cut-off distance for long-range interactions of 12 Å were also set.

Thus, the solvated system was firstly relaxed with a 500-cycle long unrestrained minimization step followed by another 5000 cycles during which harmonic restraints were applied only to the backbone atoms of the protein with a force constant of 5 kcal/(mol Å^2^).

Later, the protein was equilibrated in a four-step protocol in which the system was gradually heated from 0 to 300 K using the Langevin dynamics model and the initial position restraints were regularly relaxed. Thus, 100-ps long MD simulation was performed using NVT conditions (*i.e.*, constant number of molecules (N), Volume (V) and Temperature (T)), restraining backbone atoms with harmonic potential of 20 kcal/(mol Å^2^) and raising the temperature from 100 to 300 K. Then, a 120-ps long heating stage from 300 to 600 K was run using NVT conditions and restraining backbone atoms with harmonic potential of 10 kcal/(mol Å^2^). Then the system was cooled for 120 ps from 600 to 300 K using NVT and restraining backbone atoms with harmonic potential of 10 kcal/(mol Å^2^). Finally, a 100-ps long simulation at 300 K was performed using NPT conditions (i.e., constant number of molecules (N), Pressure (P) and Temperature (T)) and restraining backbone atoms with harmonic potential of 5 kcal/(mol Å^2^).

After the equilibration, a 50-ns long simulation at 300 K in NVT conditions was run using minor harmonic restraints of 0.5 kcal/(mol Å^2^) applied only to backbone atoms.

Finally, the trajectory was used to identify protein surface regions that have a high propensity for ligand binding on the basis of the distribution of organic probes during the trajectory, since the frequency of probe occupation in a given area should be proportional to their binding affinity to this specific area. The positions along the last 25 ns of the simulation of each organic probe is thus integrated into a probe‐occupancy map, using the *cpptraj* module of AmberTools16, and finally visualized as a contour surface corresponding to the region most frequently sampled by each organic probe.

#### Virtual screening of putative RAS inhibitors

Two separate and subsequent in silico molecular docking experiments were applied in the screening of two datasets of putative RAS protein inhibitors with the aim of identifying and finally optimizing the hit compound.

In the first molecular docking experiment (hit identification step), a dataset of tri- and tetra-peptidomimetics was built using natural and non-natural amino acids. The *N*- and *C*-terminal parts of the compounds were enriched by different capping moieties with different sizes and polarity profiles (such as diphenylactic acid, 2-(4-*tert*-butylphenoxy)acetic acid, phenylacetic acid, 9-anthracenic acid or benzoic acid for the *N*-terminal part; and carboxamide, pyrrolidine, pyridine or 3-azopiro[5.5]undecane for the *C*-terminal part). Based on the results of substrate-binding site analysis, only positively monocharged compounds were selected and thus a library of 80,000 compounds was generated.

In the second molecular docking experiment (hit optimization step), a new peptidomimetic dataset was generated starting from P1 primary sequence by the combination of the original building blocks with specific and ad hoc alternative moieties carefully selected in order to increase either the membrane permeability and/or the receptor binding affinity of the original compound.

#### Molecular docking

The same protocol was used to perform the molecular docking experiments in both the hit identification and optimization step. The three-dimensional structures of all the putative RAS protein inhibitors were created from scratch starting from the primary sequences, parameterized using the AmberTool16 *Leap* module and ff12 AMBER force field, and finally minimized with the NAMD simulation package^61^. Parameter libraries of non-natural peptidic building blocks (if any) and capping residues were written with the AmberTool16 modules *Antechamber* and *Leap*.

The 1.35 Å resolution crystal structure of the RAS GTPase protein structure (PDB code 5P21^59^) bound to a GTP analogue (*i.e.*, GppNHp) was used as the receptor to dock all the putative RAS inhibitors. In the first preparatory step, all the water molecules as well as the co-crystallized GppNHp were removed as they were not intended to directly participate in the interaction. Thus, hydrogen atoms were added and the entire system was properly protonated using the H++ web server (http://biophysics.cs.vt.edu/H++) with default parameters^64^.

All the docking calculations was performed using SMINA, an energy minimization optimized fork of the AutoDock Vina docking program^65^. All the ligands and receptor structures were converted into input files suitable for SMINA using *prepare_ligand4.py* and *prepare_receptor4.py* scripts provided by AutoDock Tools^66^. An almost cubic grid box of 60 × 60 × 60 size with a grid space of 0.375 Å was adjusted in the RAS effectors binding region and centred around the Asp33 and Asp38 residues. Exhaustiveness, number of modes and energy range were set to 32, 100 and 50 respectively.

Once all the docking simulations had finished, the phi/psi dihedral angle Ramachandran distribution of all the building blocks, the geometry of the corresponding peptide bonds and the intermolecular contacts were calculated for the 20 top ranked docking poses. Therefore, the docking conformations incompatible with phi/psi dihedral angle Ramachandran distribution, bearing no-planar or *cis* peptide bonds or having any intramolecular contacts, were filtered out. Thus, the top ranked docking poses of each compound (if any) were merged together and sorted according to SMINA docking energy, filtering out all the compounds having a docking energy lower than − 8 kcal/mol. Then, the binding stability as well as the predicted cell membrane permeability filters (see below for details) were sequentially applied and after that, the most promising compounds were selected and underwent visual inspection.

For the final selection of peptide candidates, additional factors were taken into account (*e.g.*, peptide binding mode *consensus* with the predicted substrate binding site location and chemical properties**,** lack of close orientation of positively charged residues to hydrogen bond donors, close orientation of negatively charged residues to hydrogen bond acceptors and insertion of polar residues into highly hydrophobic clefts).

#### RAS binding stability assessment

The docking model of each compound in complex with RAS protein underwent a conjugate gradient minimization, equilibration and 3-ns long implicit solvent MD simulation, using the NAMD simulation package. Thus, the first preparatory step, the overall protonation and parameterization of the system, was performed using the *Leap* module of AmberTool16 and ff12 AMBER force field. Then, the system was relaxed with a 1000-cycle long minimization, applying harmonic restraints to all backbone atoms of the system (both receptor and ligand) with a force constant of 5 kcal/(mol Å^2^). Then, a 200-ps long equilibration step was performed by gradually heating the system to 300 K and applying harmonic restraints to the backbone atoms of the protein and the ligand with a force constant of 5 and 2 kcal/(mol Å^2^), respectively. Finally, a 3-ns long MD simulation was performed applying harmonic restraints only to the backbone atoms of the receptor protein with a force constant of 2 kcal/(mol Å^2^). In all the simulations, SHAKE^62^ was applied to restrain all bonds to hydrogen atoms while a 2-fs simulation time step and a 12-Å cutoff distance for long-range interaction were set.

Finally, a binding stability assessment was performed to compute the average root-mean-square deviation (LigRMSD_avg_) of each compound along the last 1.5 ns of the trajectory using the MolSoft ICM Browser (www.molsoft.com)^67^. Compounds with LigRMSD_avg_ values lower than 3 Å were predicted as stable binders and thus selected for the cell membrane permeability evaluation*.*

#### In silico permeability prediction

In the first preparatory step, the three-dimensional structure of each compound was created from scratch and parameterized using the AmberTool16 *Leap* module and ff12 AMBER force field.

For each compound, a set of 25 2-ns long implicit chloroform-solvated unrestrained molecular dynamics (MD) simulation was performed using the ff12 AMBER force field and NAMD simulation package. Each of the 3D-structures previously generated was firstly used to obtain a small conformational ensemble comprising a total of 25 conformers. Thus, each compound was relaxed by a short 1000-step unrestrained energy minimization and then underwent a short MD simulation conducted for 100 ps, setting the system temperature to 300 K. Finally, 25 conformers were obtained by extracting one trajectory snapshot every 4 ps and used as input for the final chloroform-solvated MD simulation.

Thus, in each simulation the system was firstly energy minimized by 5000 conjugate gradient steps. Then, the system underwent an equilibration process divided into four steps with gradual heating from 0 to 300 K for 100 ps, applying harmonic restraints with a force constant of 0.5 kcal/(mol Å^2^) on all the heavy atoms in order to preserve initial molecule geometry during heating. During the equilibration, the integration time step was set to 2 fs and the nonbonding cut-off distance to 12 Å. Finally, a production step was run, consisting of an unrestrained MD simulation conducted for 2 ns and setting the system temperature to 300 K. For each compound, all the 25 2-ns long MD trajectories were combined together and a total of 12,500 MD frames were extracted using the *cpptraj* module of AmberTools16.

Finally, for each compound the average exposure of polar atoms to the solvent during the overall simulation was extracted in terms of average polar accessible solvent area value (polASA_avg_) on the overall MD frames using the MolSoft ICM Browser. Peptides with polASA_avg_ values lower than 150 Å^2^ were predicted to be permeable by passive diffusion and thus selected for the visual inspection*.*

### Cell lines and culture

hTERT-RPE (immortalized retinal pigment epithelial human cells) and HeLa (epithelioid cervix carcinoma cells) both express RAS wild type and were obtained from the American Tissue and Cell Collection (ATCC). MPanc-96, HPAF-II, PA-TU-8902, SW1990, PA-TU 8988T and PANC-1 PDAC cell lines all express oncogenic mutated KRASG12 and were a kind gift from Prof Dr A. Kimmelman (Harvard Medical School, Boston, USA).

HeLa and PDAC cells grow in Dulbecco’s modified Eagle’s medium (DMEM) and hTERT-RPE in DMEM-HAM’s F12 (1:1) medium, both supplemented with 10% fetal bovine serum (Biological Industries, Israel), penicillin, streptomycin, and nonessential amino acids.

### Drug treatment and EGF-dependent signalling activation

Cells were seeded in a media containing 10% FBS for 24 h and were serum starved for the next 24 h. Afterwards, they were incubated with different concentrations of the compounds for 2 h. A successive treatment for 10 min with EGF (50 ng/mL) (Sigma-Aldrich) was performed in order to activate cell signalling.

### Cell transfection and plasmids

HeLa cells were transfected with pEF-HA-KRASG12V plasmid, which was kindly provided by Prof Dr R. Marais (Cancer Research UK Manchester Institute, Manchester, UK). Lipofectamine®2000 Transfection Reagent (Invitrogen) was used as a transfection method following the manufacturer’s instructions.

### SDS-PAGE, Western blot and antibodies

Proteins were resolved by SDS-PAGE and transferred onto PVDF membranes (Immobilon-P, Millipore). Non-specific binding of the antibodies was assessed by incubating the membranes for 1 h at room temperature with a buffer composed of 20 mM Tris–HCl pH 7.5, 150 mM NaCl, 0.05% Tween 20 and 5% bovine serum albumin. Protein expression was determined by probing the blots overnight at 4ºC with the specified antibodies: anti- c-RAF (BD Transduction 610151, 1:500); anti-phospho-c-RAF S338 (Cell Signaling 9427, 1:500); anti-PI3Kp110α (Cell Signaling 4249, 1:1000); anti-AKT (Cell Signaling 9272, 1:1000); anti-phospho-AKT S473 (Cell Signaling 4060, 1:1000); anti-phospho-AKT Thr308 (Cell Signaling 4056, 1:1000); anti-p44/42 MAPK (ERK1/2) (Cell Signaling 9102, 1:2000); anti-phospho-p44/42 MAPK(ERK1/2) T202/Y204 (Cell Signaling 4370, 1:2000); anti-GAP120 (Santa Cruz SC-63, 1:200); anti-HA (Sigma-Aldrich H6908, 1:1000); or anti-α-tubulin (Sigma-Aldrich T9026, 1:2000). Next, after washing the membranes they were incubated with the corresponding HRP-coupled secondary antibodies (goat anti-rabbit BioRad 170-6515 or goat anti-mouse BioRad 170–6516, 1:3000) for 60 min at room temperature and washed again. Protein detection was performed by enhanced chemiluminescence (EZ-ECL, Biological Industries). Emitted light was captioned and quantified (ChemiDoc, BioRad).

For analysis of RAS signalling, cells were lysed in a buffer containing 67 mM Tris–HCl pH 6.8 and 2% SDS and then the samples were heated at 97ºC for 15 min. After this, the protein concentration of the lysates was assayed using the Lowry method. An aliquot of 15 μg of protein per sample was loaded onto the gels. Specificity of the main antibodies used in the paper is shown in Fig. S4.

### Co-Immunoprecipitation (Co-IP)

Cells were transfected with pEF-HA-KRASG12V plasmid for 24 h and starved for the next 24 h before treatment with the peptides and EGF. Next, an IP with anti-HA antibody crosslinked to agarose beads was performed. Briefly, cells were lysed with a buffer composed of 20 mM Tris–HCl pH 7.5, 100 mM NaCl, 2 mM EDTA, 5 mM MgCl_2_, 1% (v/v) Triton X-100, 10% glycerol (v/v), 1 mM dithiothreitol (DTT), plus protease and phosphatase inhibitors (150 nM aprotinin, 20 µM leupeptin, 1 mM phenylmethylsulfonyl, 5 mM sodium fluoride and 0.2 mM sodium orthovanadate) for 10 min on ice. After clarification by centrifugation, the supernatants (500–2000 µg) were incubated with 40–50 µL of anti-HA-tag antibody crosslinked to agarose beads (clone HA-7, Sigma-Aldrich A20956) for 3 h at 4 °C under rotation. Next, the immunocomplexes obtained after 2 min of spinning at 10,000 g at 4 °C were washed and subjected to immunoblotting with the corresponding antibodies.

### RBD (Ras-binding domain of Raf-1)-pull down

RBD-pull downs were performed as previously described^68^ to determine the amount of active Ras.

### Plasmon resonance analysis

Surface Plasmon Resonance Analysis was performed by using BIAcore T200 equipment. GST-KRAS (1-166) and GST were covalently immobilized on two of the channels of CM5 Series S Chip following manufacturers instruction. KRAS was loaded either with GTP or GDP by injecting 1 mM of GTP or GDP 10 µL/min for 30 min in exchange buffer (20 mM Tris–HCl pH 7.5; 50 mM NaCl, 5% glycerol, 0.1% Triton X-100, 1 mM DTT) with 10 mM EDTA at 30ºC and loading was blocked by injecting exchange buffer with 15 mM MgCl_2_ at 10 µL/min for 5 min. P1.3, was injected in running buffer (150 mM NaCl, 50 mM Tris–HCl pH7.5, 2 mM MgCl2, 0.1% Triton and 5%DMSO), at a flow rate of 60 μL/min for 1 min, at 25 °C. Dissociation was allowed for 10 min in the same buffer. All runs were done by duplicate. Nonspecific binding was subtracted by using two linked channels (GST-KRAS minus GST). Diverse solutions (from 3 to 8%) with DMSO were also prepared to analyse its effect in the RUs, and a solvent correction was performed to reduce the error associated with the injection of the sample.

### Cell viability assay

10,000 cells in 50 μL of 10% FBS-containing medium, were cultured for 24 h and then treated with the drugs (50 μL final volume) for a further 24 h in each well of a 96-well plate (100 µL final volume). MTS viability assay (CellTiter 96® Aqueous One Solution Cell Proliferation Assay, Promega G3580) was performed following the manufacturer’s specifications. The absorbance of each well was measured with a multimode plate reader (Spark, Tecan) at 490 nm. The percentage of cell viability was calculated by dividing the absorbance of each well by the average absorbance of the control wells (which had no significant deviation when Students’ T-Test was applied).

## Supplementary Information

Supplementary Figures.

Supplementary Tables.
