# Supplementary material for: Peptidomimetics designed to bind to RAS effector domain are promising cancer therapeutic compounds
Source: Sci Rep. 2022 Sep 22;12:15810. doi: 10.1038/s41598-022-19703-6 (PMC9499927; doi:10.1038/s41598-022-19703-6)

**Peptidomimetics designed to bind to RAS effector domain are promising cancer therapeutic compounds**

Chiara Pallara, Debora Cabot, Josep Rivas, Sonia Brun, Jesús Seco, Baraa Abuasaker, Teresa Tarragó, Montserrat Jaumot, Roger Prades, Neus Agell*

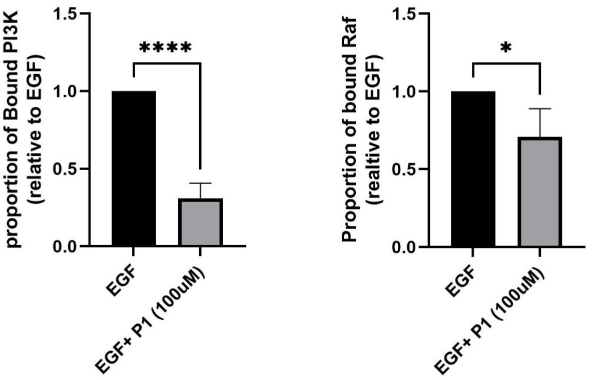


**Figure S1 Quantifications of the coimmunoprecipitation of PI3K and CRAF with KRAS in cells treated with EGF plus or minus 100 µM P1**. Quantification correspond to 3 different experiments performed as the one shown in figure 3d.

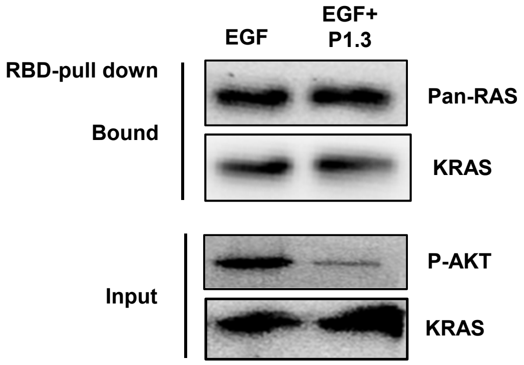


**Figure S2. P1.3 treatment does not have an effect on RAS-GTP levels**. Serum starved RPE cells were preincubated or not with 25 µM P1.3 for 2h and then treated for 10 min with EGF. Cells were lysed and the RAS-GTP levels determined by RBD-pull down as indicated in the methods section. P-AKT was also analyzed in the not bound to confirm the inhibitory activity of P1.3 in this specific experiment.

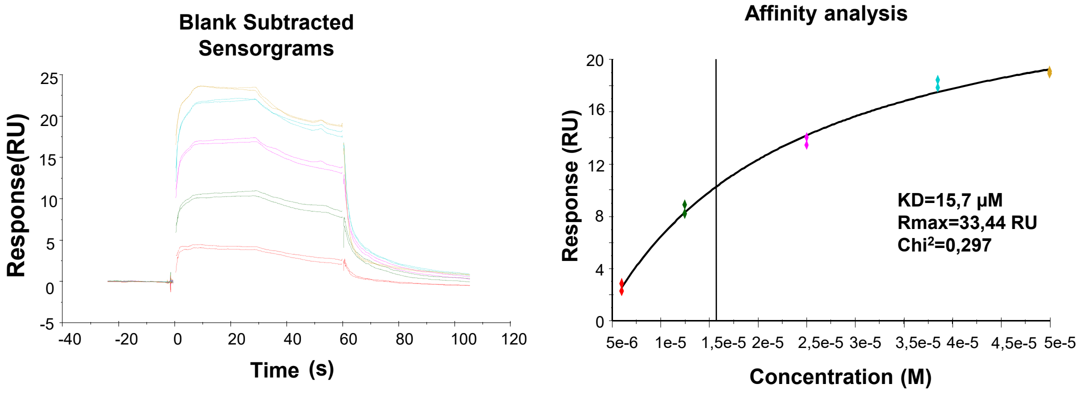


**Figure S3. Direct interaction between KRAS-GST and P1.3 determined by Plasmon Resonance.** Sensorgrams (left) and affinity analysis (right) determined as indicated in the methods. (KD: dissociation constant). Colors in upper and lower graph correspond to the same concentrations.

1. hTERT-RPE serum starved cells
2. hTERT-RPE serum starved cells + EGF 10 min
3. hTERT-RPE serum starved cells + EGF 10 min (preincubated 2h with 25µM P1.3 )

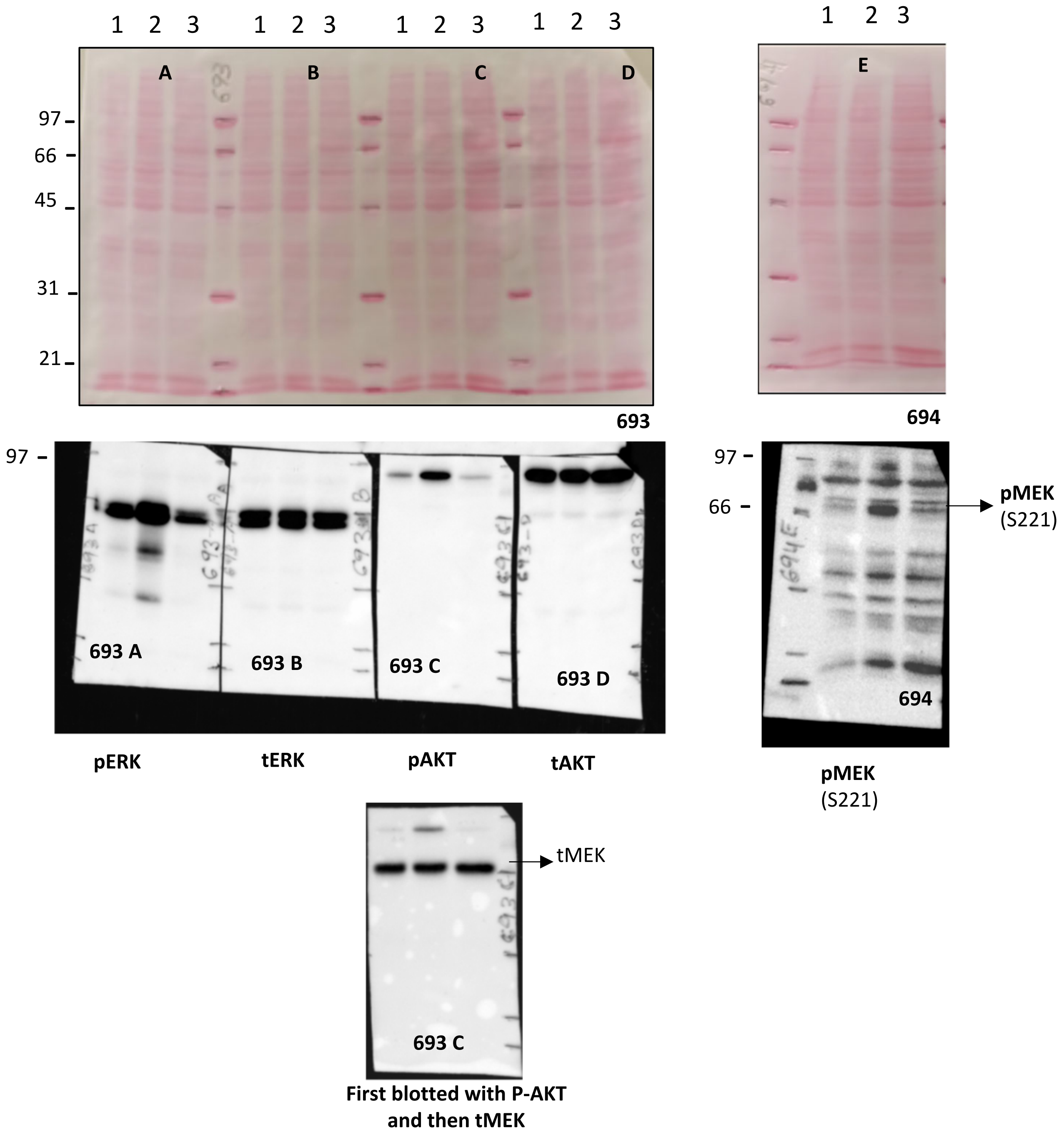


**Figure S4** Images of WB and Ponceau staining of the membranes to show the specificity of the main antibodies used in the work. The same indicated samples were loaded 5 times and the membranes blotted with the specified antibodies. The only antibody that shows unspecific bands is P-MEK, but the specific band is clearly identified.

## Merged membranes and blots corresponding to figures 3, 5 and S2

### Correspoding to fig 3a

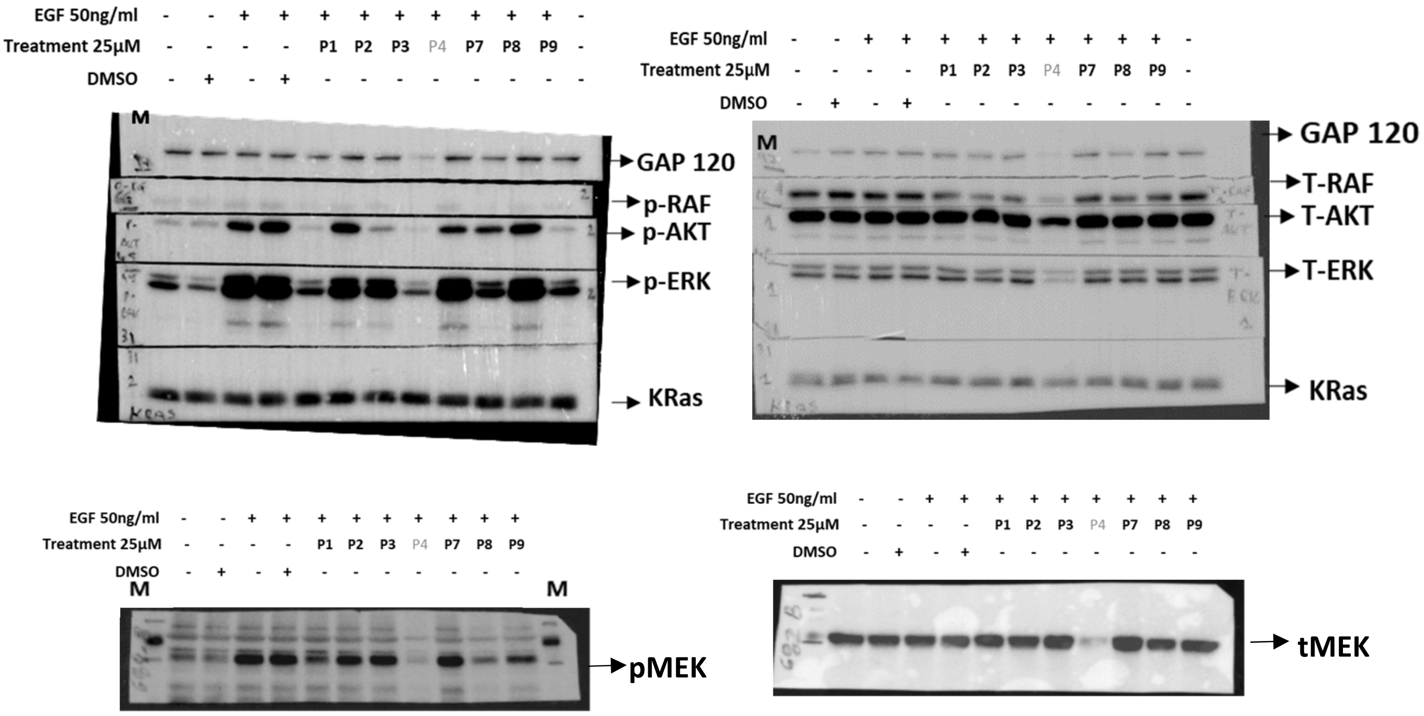


### Correspoding to fig 3b

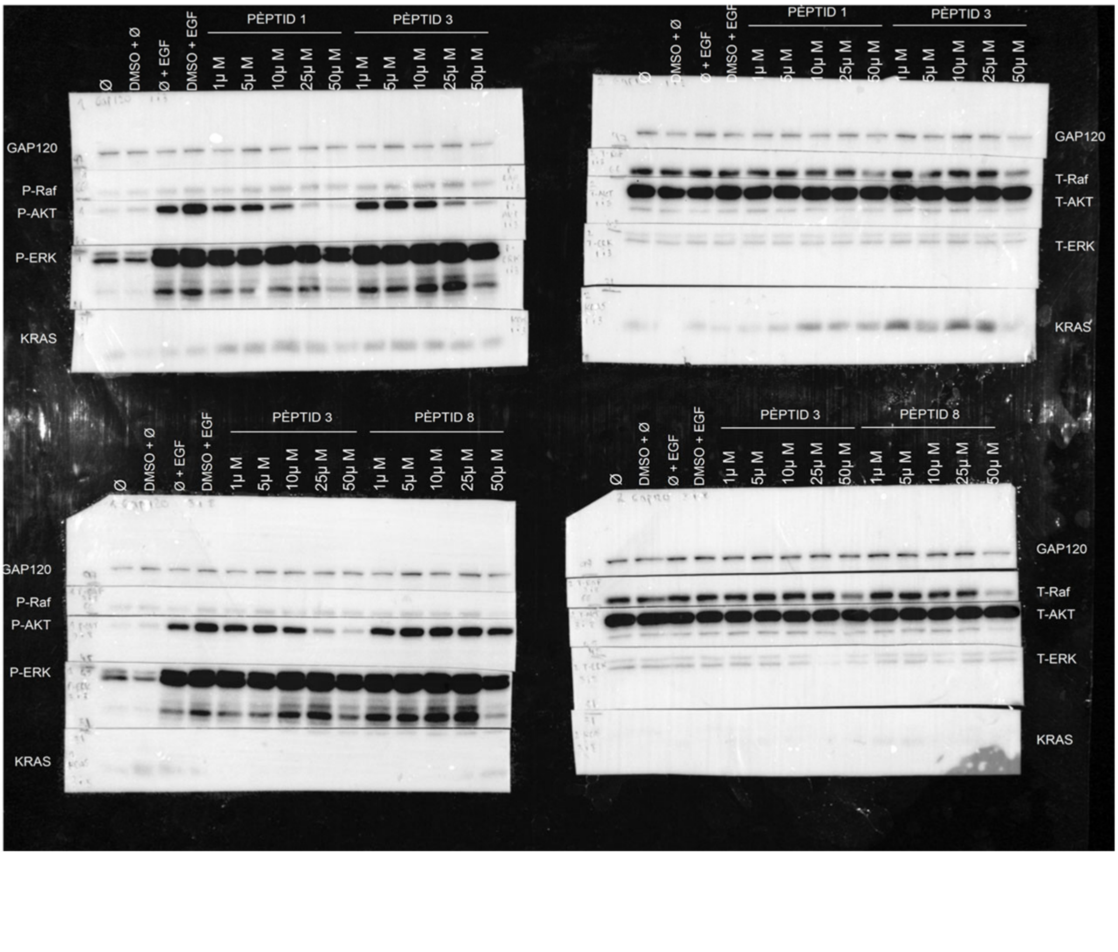

## Correspoding to fig 3c

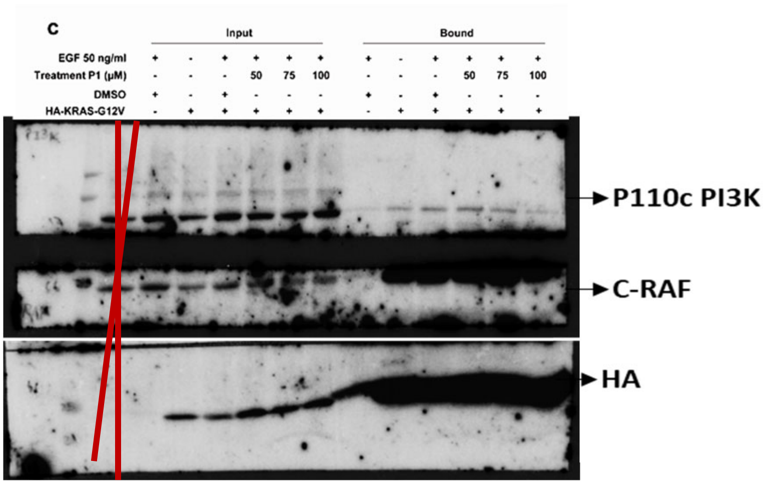


+exposed

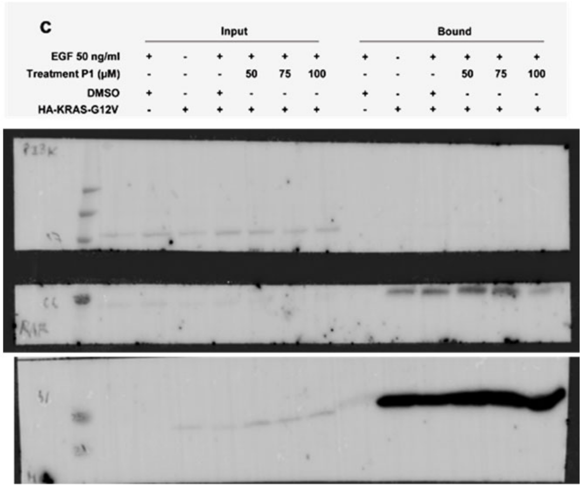


-exposed

## Correspoding to fig 3d

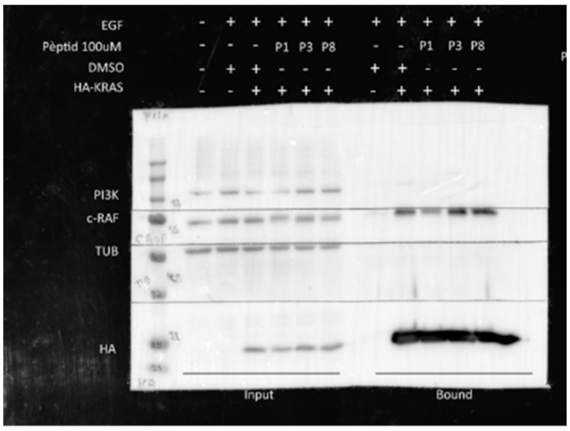


-exposed

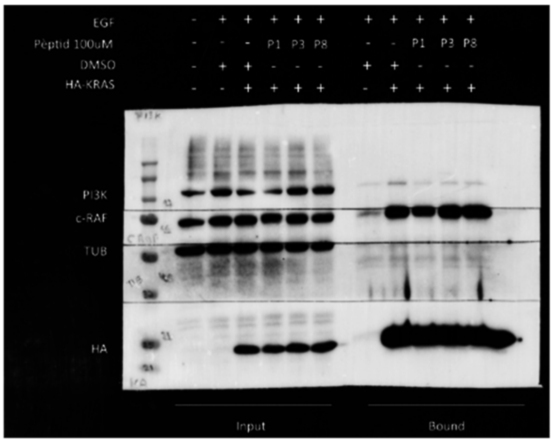


+exposed

## Correspoding to fig 5a

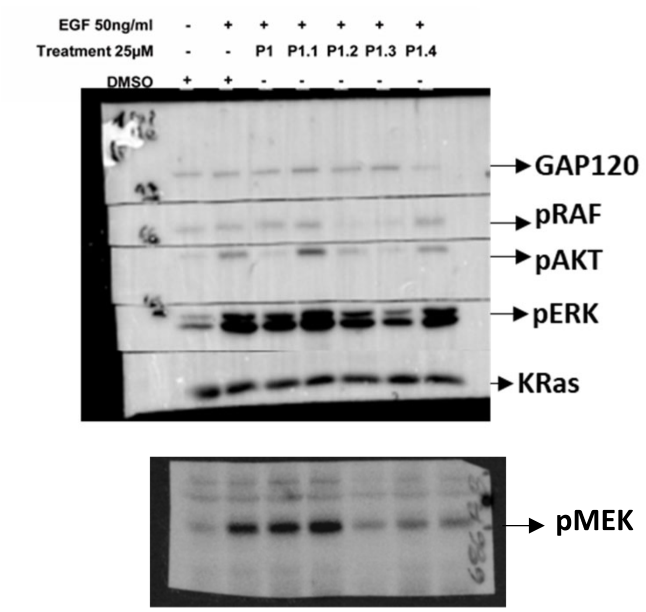


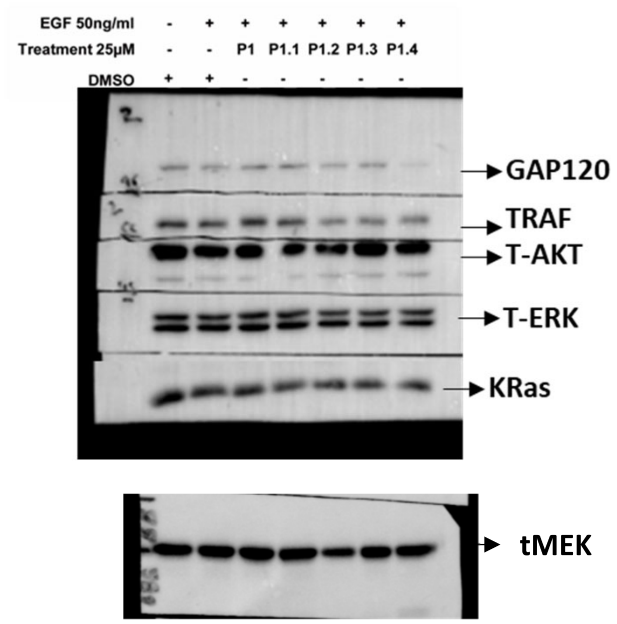


## Corresponding to fig S2

**RBD-Bound**

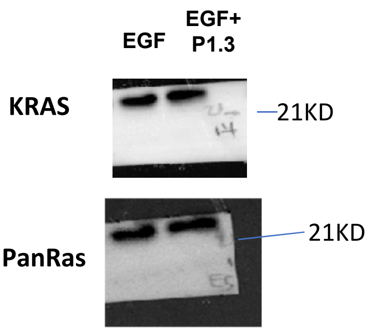


**inputs**

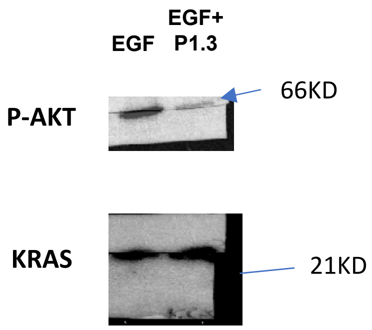

Supplement: Supplementary file 1 — Supplementary Figures. [file 41598_2022_19703_MOESM1_ESM.pdf]
