# Supplementary material for: Peptidomimetics designed to bind to RAS effector domain are promising cancer therapeutic compounds
Source: Sci Rep. 2022 Sep 22;12:15810. doi: 10.1038/s41598-022-19703-6 (PMC9499927; doi:10.1038/s41598-022-19703-6)
Supplement: Supplementary file 2 — Supplementary Tables. [file 41598_2022_19703_MOESM2_ESM.pdf]

**S1 Table 1. All available RAS-effector complex structures**

| PDB code | Method | Resolution (Å) | Ras isoform | RAS effector |
|---|---|---|---|---|
| 4G0N | X-ray | 2.45 | H-RAS | CRAF |
| 4G3X | X-ray | 3.25 | H-RAS | CRAF |
| 3KUD | X-ray | 2.15 | H-RAS | CRAF |
| 1K8R | X-ray | 3.00 | H-RAS | BYR2 |
| 2MSE | NMR | - | K-RAS | ARAF |
| 1LFD | X-ray | 2.1 | H-RAS | RALGDS |
| 1HE8 | X-ray | 3.00 | H-RAS | PI3K |
| 2C5L | X-ray | 1.9 | H-RAS | PLCε |
| 3DDC | X-ray | 1.8 | H-RAS | RASSF |
| 4K81 | X-ray | 2.4 | H-RAS | GRAB14 |
